# The Incidence Rate and Risk Factors of Malignancy in Elderly-Onset Inflammatory Bowel Disease: A Chinese Cohort Study From 1998 to 2020

**DOI:** 10.3389/fonc.2021.788980

**Published:** 2021-12-09

**Authors:** Zheng Wang, Huimin Zhang, Hong Yang, Mengmeng Zhang, Jiaming Qian

**Affiliations:** Department of Gastroenterology, Peking Union Medical College Hospital, Chinese Academy of Medical Sciences and Peking Union Medical College, Beijing, China

**Keywords:** elderly-onset inflammatory bowel disease, incidence rate, malignancies, risk factor, cohort study

## Abstract

**Background:**

Patients suffering from inflammatory bowel disease (IBD) have an increased risk of cancer. However, the risk of malignancy in patients with elderly-onset IBD (≥60 years) remains controversial. Hence, we aimed to identify and compare the dissimilarities in morbidity and related risk factors between patients with elderly-onset and adult-onset (18–59 years) IBD in a Chinese cohort.

**Methods:**

Patients with confirmed IBD, diagnosed at age ≥18 years, between January 1998 and December 2020 at the Peking Union Medical College Hospital were enrolled. The yearly incidence rates (IRs) for cancer were calculated, and the characteristics were analyzed in these patients.

**Results:**

A total of 1,480 patients suffering from adult-onset IBD and 129 patients suffering from elderly-onset IBD with a median follow-up period of 4.9 years and 4.8 years, respectively, were included. Patients in the elderly-onset IBD group demonstrated an increased overall incidence of cancer than that demonstrated by patients in the adult-onset group (IR 26.9 versus 9.51, respectively, per 1,000 person-years; relative risk [RR], 2.83). Colorectal cancer was the most common malignancy in the two groups, and patients suffering from elderly-onset IBD demonstrated a higher incidence of the malignancy (IR, 7.07 versus 3.34, respectively, per 1,000 person-years; RR, 2.12). Among the extraintestinal cancers, hematological malignancies and urinary tract cancers (including renal and urinary bladder carcinoma) were common in the elderly-onset group (IR, 4.24 and 4.24 per 1,000 person-years, respectively), whereas thyroid cancer was more common in the adult-onset group (IR, 1.36 per 1,000 person-years). Analysis of clinical characteristics revealed that patients with elderly-onset IBD who developed cancer were more likely to have diabetes and urinary lithiasis (*p* = 0.041 and 0.035, respectively). In addition, patients in the elderly-onset group had a shorter course from IBD to cancer, less exposure to immunosuppressants, less extraintestinal manifestations, and higher cancer-related mortality. Cox proportional risk regression analysis in the elderly-onset IBD group revealed that diabetes was an independent risk factor for the progression to cancer (hazard ratio [HR], 12.53 [2.379–65.994], *P* = 0.003).

**Conclusion:**

The risk of malignancy in patients suffering from elderly-onset IBD increased significantly as compared with those with adult-onset disease. Therefore, cancer monitoring should be initiated earlier for patients in the elderly-onset group.

## Introduction

Inflammatory bowel disease (IBD), comprising Crohn’s disease (CD) and ulcerative colitis (UC), is widely accepted as a chronic inflammatory disorder of the intestine. IBD has a bimodal age of onset, with one peak between 20 and 40 years of age and another between 60 and 70 years. Diagnosis at age ≥60 years is considered elderly-onset IBD and constitutes 10%–15% of the total number of IBD cases (1). The incidence rate of elderly-onset IBD has been reported as 4–8/100,000 person-years (2). However, with the aging population in China, the burden and incidence of elderly-onset IBD continue to rise. Data obtained from Hong Kong revealed that the incidence of elderly-onset UC increased from 0.1 per 100,000 persons (before 1991) to 1.3 per 100,000 persons after 2010 (3, 4). In addition, it is known that the clinical characteristics of the patients suffering from elderly-onset and adult-onset IBD (18–59 years) are not always consistent. However, sufficient attention has not been paid to elderly-onset IBD owing to its relatively lower incidence in China.

IBD, as a chronic disease, is prone to be associated with various comorbidities and/or complications throughout its course. A study conducted in Canada reported that 785.6 per 100,000 patients with IBD developed malignancies each year (5). The factors associated with IBD-related malignancies include the location of the lesion, duration, medications, and special comorbidities. Patients with IBD are especially at an elevated risk of developing colitis-associated cancers, which are related to the extent of inflammation, severity, and course of the disease (6). Studies have demonstrated that chronic inflammatory conditions can lead to malignancies even in other organs (7). In addition, IBD-related therapeutic drugs may alter the immune status, which in turn has a profound impacts on tumorigenesis. For example, azathioprine and anti-TNFα biologics have been mentioned to be a risk factor for hematologic malignancies in patients suffering from IBD (8, 9). Recent studies have shown that certain comorbidities including primary sclerosing cholangitis, chronic kidney disease, respiratory disease, and diabetes mellitus are associated with carcinogenesis in IBD patients (HR, 2.43; OR = 1.29, 1.07, and 1.06, respectively) (5, 10). The high risk of malignancy in patients with IBD has been reported as SIR of 1.2–1.6 (11–13). Data obtained from Hong Kong revealed a significant increase in the incidence of colorectal cancer in patients of elderly-onset UC as compared with those of non-elderly onset UC (0.9% versus 3.2%, respectively, *p* = 0.033) (3). There is evidence that clinical characteristics including complications and medications used differ between patients suffering from elderly-onset and non-elderly-onset IBD. In addition, a study reported that later onset of IBD was associated with a higher risk of early colorectal cancer (14). However, recent cohort studies conducted in the Western populations have shown that elderly-onset IBD only leads to an increased risk of extraintestinal tumors (7, 15), and there is no difference in the risk of colorectal cancer compared to patients with adult-onset disease. Furthermore, studies have shown that corticosteroids increase the cancer risk in patients of elderly-onset IBD (16, 17).

IBD is an emerging disease, the incidence of which is increasing rapidly in China, and several patients are developing complications, including cancer. It is known that elderly patients are vulnerable to cancer. Therefore, research on the incidence and risk factors of malignancy in China is essential to develop preventive strategies and contribute to the global data. The differentiation between elderly patients with IBD and those with elderly-onset IBD has been suboptimal in previous studies. Here, we attempted to identify the dissimilarities in the incidence of cancer between patients with elderly-onset IBD and adult-onset IBD in a Chinese cohort. We aimed to provide information to develop cancer-monitoring strategies in patients of elderly-onset IBD.

## Methods

### Study Design

All data for this cohort study were obtained from the medical documents, telephonic follow-up records, and the National Central Cancer Registry database from January 1998 to December 2020 of patients diagnosed with or hospitalized for the treatment of IBD at the Peking Union Medical College Hospital. The Ethics Review Committee of the Peking Union Medical College Hospital approved this study.

### Study Population

Inclusion criteria: (1) patients diagnosed with IBD (UC and CD) based on the third European Crohn’s and Colitis Organization consensus guidelines and (2) IBD diagnosed at age ≥18 years.

Exclusion criteria: (1) patients with a history of malignancy before the diagnosis of IBD; (2) patients with a previous history of a specific autoimmune disease; the reason for exclusion was baseline claims for biologics, immunomodulators, or corticosteroids; (3) patients with unclassified IBD were excluded owing to unclassified disease severity and extent; and (4) patients without ≥1 day of follow-up. Patients were regularly (at least once a year for most patients) followed up, from the date of diagnosis of IBD until the outcome occurrence, death, or terminal point of the study (June 2021).

### Data Collection

Patients with cancer occurring after the diagnosis of IBD and during follow-up were included. The retrospectively collected data at diagnosis included sex, age at IBD diagnosis, type of IBD, disease extent, the behavior of CD, intestinal complications (including fistula, stenosis, obstruction, perforation, bleeding, abdominal abscess, perianal lesions, and toxic megacolon), extraintestinal manifestations (oral ulcers, skin lesions, joint lesions, ocular lesions, fatty liver, cholelithiasis, and thrombotic disease), comorbidities (diabetes mellitus, urolithiasis, hypertension, coronary heart disease, myocardial infarction, and heart failure), and a history of alcohol abuse and smoking. The data recorded during follow-up included drug exposure (5-aminosalicylic acid, glucocorticoids, azathioprine, methotrexate, thalidomide, and biologics), the outcome of cancer, age at diagnosis of cancer, and cause of death.

### Outcome Measures

The time to the diagnosis of cancer was the primary outcome measure. The diagnosis of cancer was based on pathological features. The date of diagnosis, type of cancer, affected organ, and histological type were recorded.

The incidence of cancer and mortality was estimated as the incidence densities, which were calculated as the number of new cancer cases or death divided by the number of patient-years (18). The incidence of cancer between 1998 and 2008 has been presented as the accumulated incidence rate considering the small number of enrolled cases and cancer cases. The annual incidence of cancer for the entire cohort was the ratio of the number of new cancer cases (from January 1 to December 31 of each year) to the number of person-years at risk (5), which were calculated for the eligible patients from January 1 to the last data collection, cancer occurrence, death, or endpoint of the year (December 31) (18). Poisson regression was used to measure the annual incidence rates of cancer in the adult-onset and elderly-onset groups, which have been presented graphically as 3-year centered moving averages from 2009 to 2019 (5).

### Statistical Analysis

Data are presented as means and standard deviations (SD), medians and interquartile ranges (IQRs), or frequencies and percentages. IBM SPSS version 21 (Armonk, NY, USA) and GraphPad Prism version 7.0 (San Diego, CA, USA) were used for data analyses. The Mann–Whitney *U* test or Fisher’s exact test was performed to analyze differences between the two groups. Cox regression and logistic regression analysis were used for multivariate analysis.

## Results

### Cohort Description

**Figure 1:**
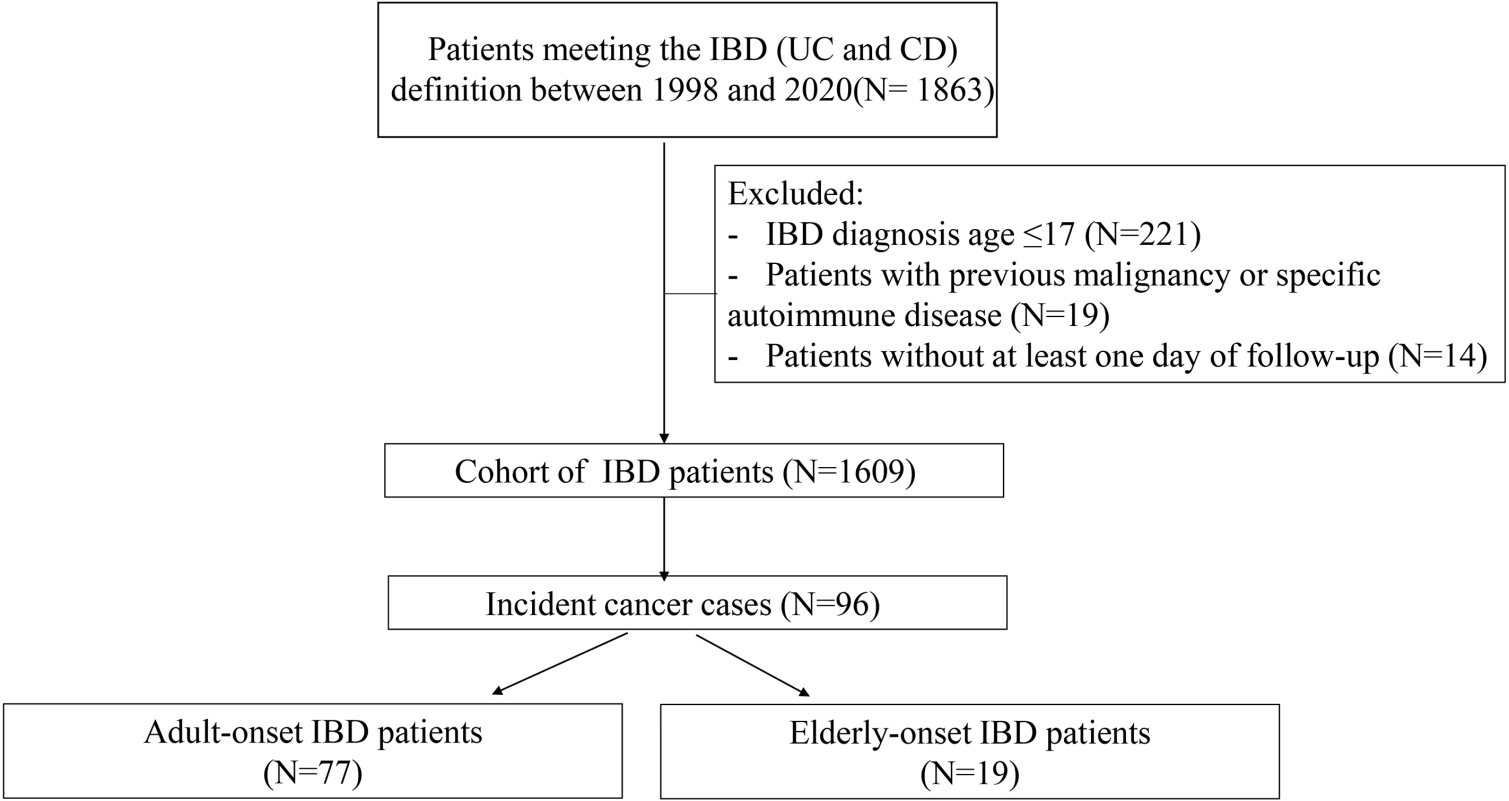
Cohort definition and flowchart.

A total of 1,863 patients with confirmed IBD from January 1998 to December 2020 were identified, and 254 cases were excluded according to the aforementioned criteria (**Figure 1**). Finally, 1,609 patients were included (1,065 with UC and 544 with CD), and the total follow-up summation was 8,799.94 person-years. Patients were categorized into the adult-onset IBD (18–59 years) or elderly-onset IBD (≥60 years) group based on their age at diagnosis, with the median follow-up of 4.9 years and 4.8 years, respectively, and the total follow-up duration of 8,092.9 years and 707.1 years, respectively. Among the 1,480 patients with adult-onset disease (966 with UC and 514 with CD) and 129 patients with elderly-onset disease (99 with UC and 30 with CD), the mean age at diagnosis of IBD was 35.5 (SD, 11.4) years and 65.2 (SD, 5.63) years (*p* < 0.0001), respectively, and men accounted for 59.1% and 65.1% of the cases, respectively. Moreover, 17.1% of the patients with elderly-onset IBD had a long-term history of smoking (>35 years), and 13.2% had a history of heavy drinking (>50 g/day on average for men and >25 g/day for women). Diabetes, hypertension, and coronary heart disease were more common among patients in the elderly-onset IBD (*p* = 0.001, <0.0001, and <0.0001, respectively), as shown in **Table 1**.

**Table 1:** Patient characteristics of the cohort.

	Adult-onset IBD (Age 18–59)	Elderly-onset IBD (Age ≥60)	*p*-value
Total patients (*N*)	1,480	129	0.007
UC	966 (65.3)	99 (76.7)	
CD	514 (34.7)	30 (23.3)	
Sex, *N* (%)			0.219
Male	874 (59.1)	84 (65.1)	
Female	606 (40.9)	45 (34.9)	
Age at IBD diagnosis			<0.0001
Mean (SD)	35.5 (11.4)	65.2 (5.63)	
Follow-up time, years			0.985
Mean (SD)	5.46 (4.18)	5.46 (4.28)	
Median (IQR)	4.85 (5.36)	4.79 (6.19)	
Smoke duration, *N* (%)			<0.0001
Never	1,107 (74.8)	76 (58.9)	
<15 years	174 (11.8)	6 (4.65)	
15–35 years	163 (11.0)	25 (19.4)	
>35 years	36 (2.4)	22 (17.1)	
Drink, *N* (%)			<0.0001
Never	1,077 (72.8)	78 (60.5)	
Mild	271 (18.3)	25 (19.4)	
Moderate	65 (4.40)	9 (6.98)	
Severe	67 (4.50)	17 (13.2)	
Appendectomy history, *N* (%)	111 (7.50)	7 (5.43)	0.372
Comorbidities, *N* (%)			
Diabetes	63 (4.30)	14 (10.9)	0.001
Hypertension	125 (8.45)	55 (42.6)	<0.0001
Coronary disease	38 (2.57)	22 (17.1)	<0.0001
Urolithiasis	840 (56.8)	83 (64.3)	0.123

### Incidence Trends of Malignancy in Patients With Elderly-Onset and Adult-Onset IBD

**Figure 2:**
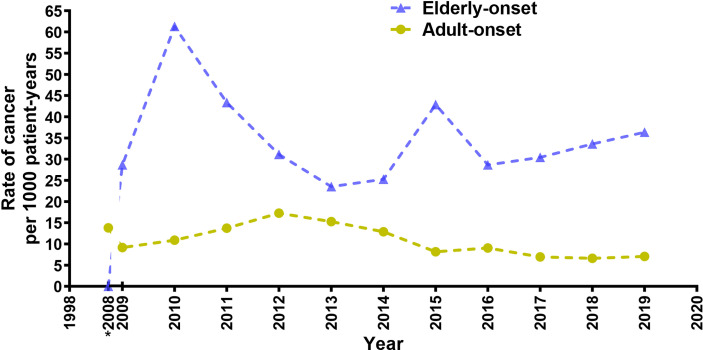
Annual incidence rate of cancer for elderly-onset IBD and adult-onset IBD as a function of calendar time presented as 3-year moving averages from 2009 to 2019. *Accumulated incidence rate of cancer from 1998 to 2008.

**Figure 3:**
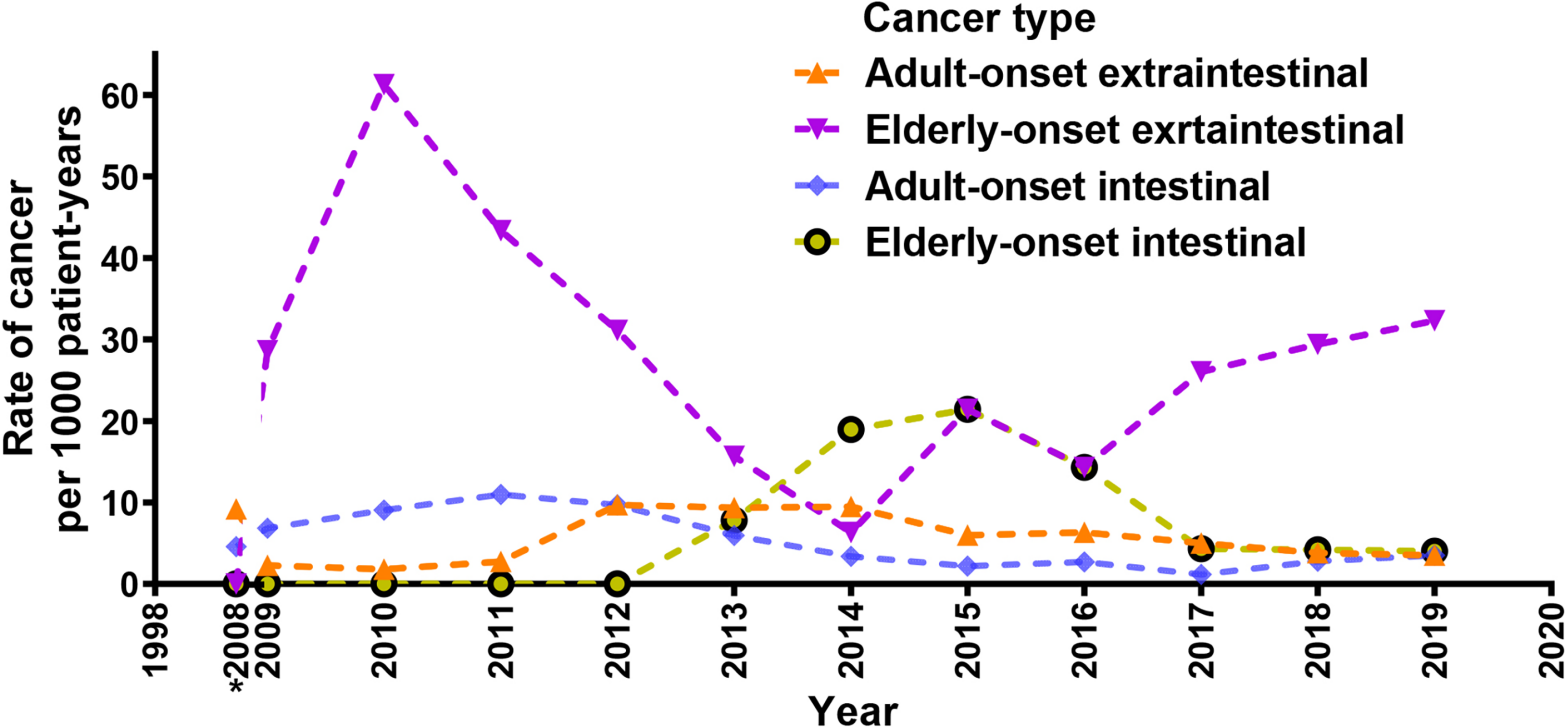
Crude rates of elderly-onset IBD and adult-onset who developed intestinal or extra-intestinal cancers presented as 3-year moving averages from 2009 to 2019. *Accumulated incidence rate of cancer from 1998 to 2008.

A total of 96 cancer cases occurred during the study (10.9 per 1,000 person-years; 95% confidence interval [CI], 8.9 to 13.3 per 1,000 person-years), including 77 cases in the adult-onset and 19 cases in the elderly-onset IBD groups. The incidence was significantly higher among patients in the elderly-onset group than in the adult-onset group (26.9 versus 9.51 per 1,000 person-years, respectively; RR, 2.83). Among the intestinal tumors, the incidence of colorectal cancer in the elderly-onset group was also higher than that in the adult-onset group (7.07 versus 3.34 per 1,000 person-years, respectively; RR, 2.12). In terms of extraintestinal cancers, thyroid cancer was the most common in patients with adult-onset IBD (14.3%, 1.36 per 1,000 person-years; 95% CI, 0.8–2.5 per 1,000 person-years), followed by hematological tumors and cervical malignancies (including cervical and endometrial cancers; 9.10%, 0.87 per 1,000 person-years; 95% CI, 0.4–1.8 per 1,000 person-years). However, hematological and urinary tract tumors (including kidney and bladder cancer) were the most common among patients in the elderly-onset IBD group (15.8%, 4.24 per 1,000 person-years; 95% CI, 1.4–12.3 per 1,000 person-years), as shown in **Table 2**. As no malignancy was recorded among patients with elderly-onset IBD from 1998 to 2008 (shown in **Supplementary Table 1**), the incidence rate of cancer between 1998 and 2008 has been presented as the accumulated incidence rate and annual incidence of malignancy in the two groups were analyzed from 2009 to 2019. As shown in **Figure 2**, the overall annual incidence of tumors in the elderly-onset IBD group was always above the morbidity in the adult-onset group. Further stratification of tumors showed that the incidence of extraintestinal cancers in the elderly-onset group was also beyond that of the adult-onset group (except in 2014) and showed an increasing trend after 2016. However, the incidence of intestinal tumors gradually decreased from 2015. The incidence of extraintestinal tumors in the adult-onset group showed a decreasing trend; however, that of intestinal tumors showed an increasing trend after 2017, as shown in **Figure 3**.

**Table 2:** Distribution and incidence rates of malignancy among adult-onset and elderly-onset groups.

	Adult-onset IBD (Age 18–59)		Elderly-onset IBD (Age ≥60)	
	*N* (%)	Per 1,000 PYs [95% CI]	*N* (%)	Per 1,000 PYs [95% CI]
GI malignancies	32 (41.6)	3.95 [2.80–5.60]	8(42.1)	11.3 [5.70–22.1]
Colorectal cancer	27 (35.1)	3.34 [2.30–4.80]	5(26.3)	7.07 [3.00–16.5]
SBA	1 (1.30)	0.12 [0–0.70]	0	0
Appendiceal mucinous neoplasms	2 (2.60)	0.25 [0–0.80]	0	0
Hepatobiliary malignancy	2 (2.60)	0.25 [0–0.80]	3(15.8)	4.24 [1.4–12.3]
Lung cancer	5 (6.49)	0.62 [0.3–1.4]	2(10.5)	2.83 [0.80–10.2]
Urinary tract malignancy	5 (6.49)	0.62 [0.3–1.4]	3(15.8)	4.24 [1.4–12.3]
Hematological malignancy	7 (9.10)	0.86 [0.4–1.8]	3(15.8)	4.24 [1.4–12.3]
Thyroid cancer	11 (14.3)	1.36 [0.8–2.5]	0	0
Genital malignancy				
Female breast cancer	5 (6.49)	0.62 [0.3–1.4]	1(5.26)	1.41 [0.20–7.90]
Prostate cancer	1 (1.30)	0.12 [0–0.70]	2(10.5)	2.83 [0.80–10.2]
Uterus malignancy	7 (9.10)	0.87 [0.4–1.8]	0	0
Ovarian cancer	2 (2.60)	0.25 [0–0.80]	0	0
Others	2 (2.60)	0.25 [0–0.80]	0	0
Total	77 (100)	9.51 [7.6–11.9]	19(100)	26.9 [17.3–41.6]

PYs, patient-years; SBA, small bowel adenocarcinoma.

### Characteristics of IBD Patients Who Developed Malignancies

Univariate analysis of IBD and cancer cases revealed that the time from IBD onset to tumor development in the elderly-onset group was significantly shorter than that in the adult-onset group (4.28 ± 4.15 versus 12.1 ± 8.75, respectively, *p* < 0.0001). In addition, the cancer-related mortality rate was higher in the elderly-onset group than in the adult-onset group (8.45 versus 0.865 per 1,000 person-years; 31.5% versus 9.72%, respectively; *p* = 0.007). Patients with cancer in the elderly-onset IBD group had a long-term history of smoking (>35 years) than those in the adult-onset group (26.3% versus 5.2%, respectively, *p* = 0.03). Regarding comorbidities, the proportion of sufferers with diabetes or urinary calculi in the elderly-onset group was distinct compared with the adult-onset group (15.8% versus 2.6%, respectively; *p* = 0.041; 78.9% versus 53.2%, respectively; *p* = 0.035). Moreover, cancer cases in the elderly-onset group showed a significantly lower rate of extraintestinal manifestations, including joint pain and oral ulcers (0 versus 23.4%, respectively; *p* = 0.003; 0 versus 24.7%, respectively; *p* = 0.002), and the incidence of perianal lesions and intestinal obstruction was also significantly lower (0 versus 15.6%, respectively; *p* = 0.017; 0 versus 14.3%, respectively, *p* = 0.03). Regarding the medication history, the use of glucocorticoids, azathioprine, and thalidomide among cancer patients in the elderly-onset IBD group was not common (*p* = 0.027, 0.003, and 0.030, respectively), and the exposure rates of three or more IBD drugs were 0% versus 27.6%, respectively (*p* = 0.001), as shown in **Table 3**.

**Table 3:** Patient characteristics of adult-onset and elderly-onset IBD patients who developed a malignancy.

	Adult-onset IBD (Age 18–59)	Elderly-onset IBD (Age ≥60)	*p*-value
Total patients (*N*)	77	19	
Sex, *N* (%)			0.071
Male	39 (51.9)	14 (73.7%)	
Female	38 (48.1)	5 (26.3%)	
IBD diagnosis, *N* (%)			0.279
UC	61 (79.2)	17 (89.5)	
CD	16 (20.8)	2 (10.5)	
Age at IBD diagnosis			<0.0001
Mean (SD)	37.5 (10.6)	66.6 (4.06)	
Median(IQR)	37 (17.00)	66 (5.0)	
Duration of disease to cancer, years			<0.0001
Mean (SD)	12.1 (8.75)	4.28 (4.15)	
Median(IQR)	11.3 (12.3)	3.53 (3.39)	
Death, *N* (%)	8 (11.1)	7 (36.8)	0.007
Cancer-related death	7 (9.72)	6 (31.5)	
Others	1 (1.39)	1 (5.26)	
Family history of cancer	22 (28.6)	4 (21.1)	0.5
Smoke duration, *N* (%)			0.03
Never	61 (79.2)	13 (68.4)	
<15 years	7 (9.1)	0	
15–35 years	5 (6.5)	1 (5.3)	
>35 years	4 (5.2)	5 (26.3)	
Drink, *N* (%)	16 (20.8)	5 (26.3)	0.437
Appendectomy history, *N* (%)	6 (7.8)	0	0.098
Comorbidities, *N* (%)			
Diabetes	2 (2.6)	3 (15.8)	0.041
Hypertension	20 (26.3)	8 (42.1)	0.187
Coronary disease	6 (7.8)	4 (21.1)	0.117
Urolithiasis	41 (53.2)	15 (78.9)	0.035
IBD-related surgery, *N* (%)	12 (15.6)	4 (21.1)	0.576
Extra-intestinal manifestation			
Arthralgia	18 (23.4)	0	0.003
Oral ulcer	19 (24.7)	0	0.002
Eye lesion	4 (5.2)	0	0.179
Fatty liver	7 (7.8)	2 (10.5)	0.850
Cholelithiasis	6 (7.8)	2 (10.5)	0.707
Complication			
Bleeding	11 (14.3)	1 (5.3)	0.246
Perforation	4 (5.2)	0	0.179
Obstruction	11 (14.3)	0	0.03
Skin lesion, *N* (%)	4 (5.2)	0	0.179
Perianal lesion, *N* (%)	12 (15.6)	0	0.017
Medication exposure (ever exposed)			
5-ASA	71 (92.2)	18 (94.7)	0.694
Steroids	46 (59.7)	6 (31.6)	0.027
Thiopurines	18 (23.4)	0	0.003
Methotrexate	5 (6.50)	0	0.132
Thalidomide	10 (13.0)	0	0.030
Biologics	3 (3.90)	0	0.246
Multi-medication exposure*			0.001
0 medication	4 (5.2)	1 (5.3)	
1–2 medications	47 (65.5)	18 (93.3)	
3+ medications	26 (27.6)	0	

### Risk Factors for Malignancy in Patients With Elderly-Onset IBD

**Figure 4:**
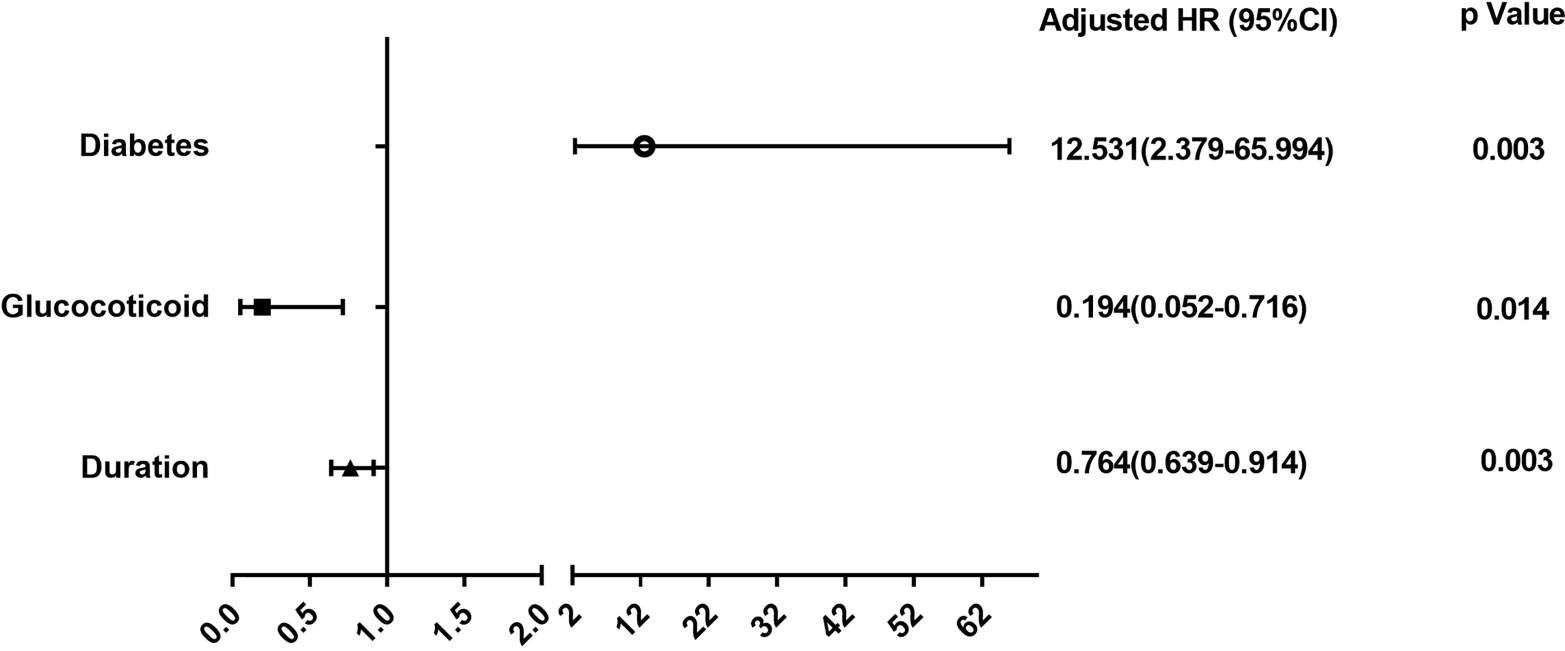
Risk factors for overall cancer in elderly-onset IBD.

We further explored the risk factors for malignancy among patients in the elderly-onset IBD using Cox regression analysis as well as multivariable logistic regression analysis adjusted for age, gender, and duration. As shown in **Figure 4** and **Supplementary Figure 1**, diabetes was a risk factor for the progression of elderly-onset IBD to malignancy (Adjusted HR, 12.53 [2.379–65.994], *p* = 0.003), whereas glucocorticoid use and the course of disease were protective factors against cancer (Adjusted HR, 0.194 [0.052–0.716], *p* = 0.014 and 0.764 [0.639–0.914], *p* = 0.003).

## Discussion

The present study focused on the incidence of malignancy in an IBD cohort in mainland China and presented several important findings. First, patients in the elderly-onset IBD group demonstrated a higher incidence of overall cancer development as compared with those in the adult-onset group, especially for colorectal cancer, which was the most common malignancy in the two groups. Among the extraintestinal cancers, lymphoproliferative or myeloproliferative disorders and urinary tract cancers were the common malignancies among elderly-onset group, whereas thyroid cancer was more common in the adult-onset group. Second, the progression of elderly-onset IBD to malignancy demonstrated a shorter course and was associated with higher tumor-related mortality. Third, diabetes mellitus was an independent risk factor for elderly-onset IBD leading to malignancy, whereas glucocorticoid use was a protective factor. Therefore, considering the aging population in China, it is necessary to initiate tumor surveillance earlier in patients with elderly-onset IBD.

In this study, the overall morbidity of malignancies in the IBD cohort from 1998 to 2020 was 10.9 per 1,000 person-years (95% CI, 8.9–13.3 per 1,000 person-years), which is higher (2.85 per 1,000 person-years) than that reported in the Hong Kong cohort (2000–2016) (13). In addition, the incidence was marginally higher than that reported by Brassard P et al. in the Western IBD population (7.86 per 1,000 person-years; 95% CI, 7.54–8.19 per 1,000 person-years) (5). To determine whether elderly-onset IBD patients had an increased risk of developing cancer compared with non-IBD elderly population, standardized incidence ratios (SIRs) with 95% confidence intervals (CIs) were calculated. Based on the national incidence rates from NCCR, we calculated the expected number of cancer cases according to gender, age, and calendar period in 1-year intervals, and compared it with the observed cancer cases from 1998 to 2020. Finally, we obtained the standardized incidence rate (SIR = 1.86 [1.063–3.021], *p* = 0.031), which further verified the role of elderly-onset IBD in increasing the overall incidence of cancer. Considering that the present study was conducted at one single center and was a hospital-based cohort, the incidence of IBD-associated malignancies could have been overestimated owing to selection bias. Therefore, a larger multicenter cohort study is required for further verification of the results. Following stratification by tumor type, IBD-related colorectal cancer constituted 33.3% of the cases, with an overall incidence of 3.6 per 1,000 person-years, which is similar to that reported in the Western countries (3.5 per 1,000 person-years) (19). Regarding extraintestinal tumors, hematological malignancies and thyroid cancer were the most common, and the high incidence of hematological malignancies in IBD is consistent with that reported in Denmark (20). Interestingly, in this cohort, all patients who developed thyroid cancer belonged to the adult-onset group. A previous large case–control study suggested that age was a protective factor against the progression of IBD to thyroid cancer (21). Radiation exposure at an early age has long been considered an independent risk factor for thyroid cancer (22). Therefore, the method of optimizing radiographical examination and reducing the incidence of thyroid cancer in young patients with IBD required further investigation. Previous studies in Western populations have reported an increased risk of extraintestinal cancers, especially prostate cancer, hematological malignancies, and skin cancer, in elderly patients with IBD (7, 15). However, the increased risk of colorectal cancer is controversial (14, 23). Our study suggested that the overall morbidity of cancer, including colorectal cancer and extraintestinal tumors (hematological, urinary, hepatobiliary, lung, prostate, and breast carcinomas), was greater in elderly-onset IBD than in adult-onset disease group (IR, 26.9 versus 9.51 per 1,000 person-years, respectively; RR, 2.83). Further analysis of the annual incidence of intestinal and extraintestinal carcinomas in both groups revealed that the incidence of parenteral tumors was higher in the elderly-onset group than in the adult-onset group. In terms of intestinal cancer, the incidence of colorectal cancer was also higher in the elderly-onset IBD group from 2013. However, the incidence started to decline after 2015 and gradually became similar to that in the adult-onset group in the last 3 years. The reason may be that colitis-associated colorectal cancer is related to the course of IBD, and the risk of malignancy in patients with adult-onset IBD is higher than that in elderly-onset patients. Therefore, the incidence of intestinal tumors in patients with younger-onset IBD may gradually increase with a longer follow-up period. Furthermore, we compared the incidence of cancer between patients in the young-onset (18–40 years old) and middle-age-onset (41–59 years old) groups; however, there was no significant difference (IR, 8.42 versus 11.8 per 1,000 person-years, respectively; *p* = 0.164) in the overall occurrence, as shown in **Supplementary Table 2**.

Analysis of clinical characteristics in the 96 cancer cases revealed that the time from IBD diagnosis to the development of malignancy was shorter, and the all-cause mortality was higher among patients in the elderly-onset group compared with that in the adult-onset group. Inflammation and aging are known to promote the development of tumors, and this synergistic effect is more significant in elderly patients with IBD, which leads to the rapid progression of carcinomas. Another interesting finding was that the proportion of diabetes mellitus in the cancer cases from elderly-onset group was significantly higher than that in the cancer cases of adult-onset IBD. The analysis of risk factors in elderly patients with IBD showed that diabetes mellitus was an independent risk factor for the progression of IBD to cancer. A recent Canadian study also suggested that diabetes mellitus could increase the incidence of IBD-related cancer (OR = 1.06; 95% CI, 1.01–1.11) (5). The underlying mechanism may be related to inflammatory mediators including IL-6, IL-1α, and TNF-α, which not only promote epithelial–mesenchymal transition through activation of the Janus kinase/signal transducer and activator of trans-ions pathway but also increase the risk of type 2 diabetes (24). This partly explains the increased risk of colorectal cancer in patients suffering from elderly-onset IBD; however, more evidence is required to support the association between diabetes, IBD, and colorectal cancer. Previously, several studies focused on the role of drug use in the cancer risk of patients with IBD and reported that glucocorticoids increased the risk of cancer (18) and azathioprine increased the risk of lymphoproliferative or myeloproliferative disorders (9). However, our study found that glucocorticoid use was a protective factor against the progression of IBD to malignancy in the elderly-onset IBD group. We hypothesize that the treatment period and dose of glucocorticoids may account for the difference. In addition, our data revealed a lower utilization rate of immunosuppressive agents (including azathioprine and thalidomide) in the elderly IBD population; therefore, the role of azathioprine in promoting the occurrence of tumors in patients with elderly-onset IBD needs to be discussed further.

To the best of our knowledge, the current work is a large sample cohort study of tumorigenesis in patients with elderly-onset IBD in mainland China, which provides an in-depth and detailed analysis of tumor incidence and related risk factors. Our study supports the view that patients with elderly-onset IBD are at a greater risk of developing cancer as compared with those with a younger-onset disease. In addition, our study indicates that early tumor onset and higher tumor-related mortality occur in this population, providing strong evidence for early surveillance in patients suffering from elderly-onset IBD. In this study, Cox regression was used for multivariate analysis. In general, multivariate analysis can be conducted by logistic regression and Cox regression. So, we used the multivariable logistic regression analysis for further verification (shown in **Supplementary Figure 1**). Not surprisingly, both statistical methods confirmed that diabetes was a risk factor for cancer in elderly-onset IBD, whereas glucocorticoid use and the course of disease were protective factors. Considering that this is a dynamic cohort, the results obtained from Cox regression analysis are displayed in **Figure 4**. The chief limitation of this study lies in the single-center design, which was limited to the inpatients treated in the Peking Union Medical College Hospital. The included patients had a relatively wide range of lesions and severe disease activity, which may have led to selection bias. Therefore, the analysis of risk factors (such as diabetes) needs to be further verified in larger multi-center cohort studies.

## Data Availability Statement

The original contributions presented in the study are included in the article/**Supplementary Material**. Further inquiries can be directed to the corresponding authors.

## Ethics Statement

The studies involving human participants were reviewed and approved by the Ethics Review Committee of the Peking Union Medical College Hospital (Ethics Review Number S-K1781). Written informed consent for participation was not required for this study in accordance with the national legislation and the institutional requirements.

## Author Contributions

ZW finished the data collection, analyzed the data, and wrote the main manuscript. JQ designed the study and revised the manuscript. HZ, HY, and MZ participated in the data collection and provided valuable suggestions. All authors contributed to the article and approved the submitted version.

## Funding

This work was funded by Health Research & Special Projects Grant of China (No.201002020 and No.201502005); CAMS Innovation Fund for Medical Sciences (No. 2016-I2M-3-001 and No.2019-I2M-2-007); National Natural Science Foundation of China (No.81570505 and No.81970495); and Natural Science Foundation of Beijing, China (No.7202161).

## Conflict of Interest

The authors declare that the research was conducted in the absence of any commercial or financial relationships that could be construed as a potential conflict of interest.

The handling editor YZ has declared a shared parent affiliation with the authors at the time of review.

## Publisher’s Note

All claims expressed in this article are solely those of the authors and do not necessarily represent those of their affiliated organizations, or those of the publisher, the editors and the reviewers. Any product that may be evaluated in this article, or claim that may be made by its manufacturer, is not guaranteed or endorsed by the publisher.
