# Supplementary material for: The Incidence Rate and Risk Factors of Malignancy in Elderly-Onset Inflammatory Bowel Disease: A Chinese Cohort Study From 1998 to 2020
Source: Front Oncol. 2021 Dec 9;11:788980. doi: 10.3389/fonc.2021.788980 (PMC8695610; doi:10.3389/fonc.2021.788980)

Supplementary Figure1-Risk factors for cancers in elderly-onset IBDs by multivariable logistic regression analysis

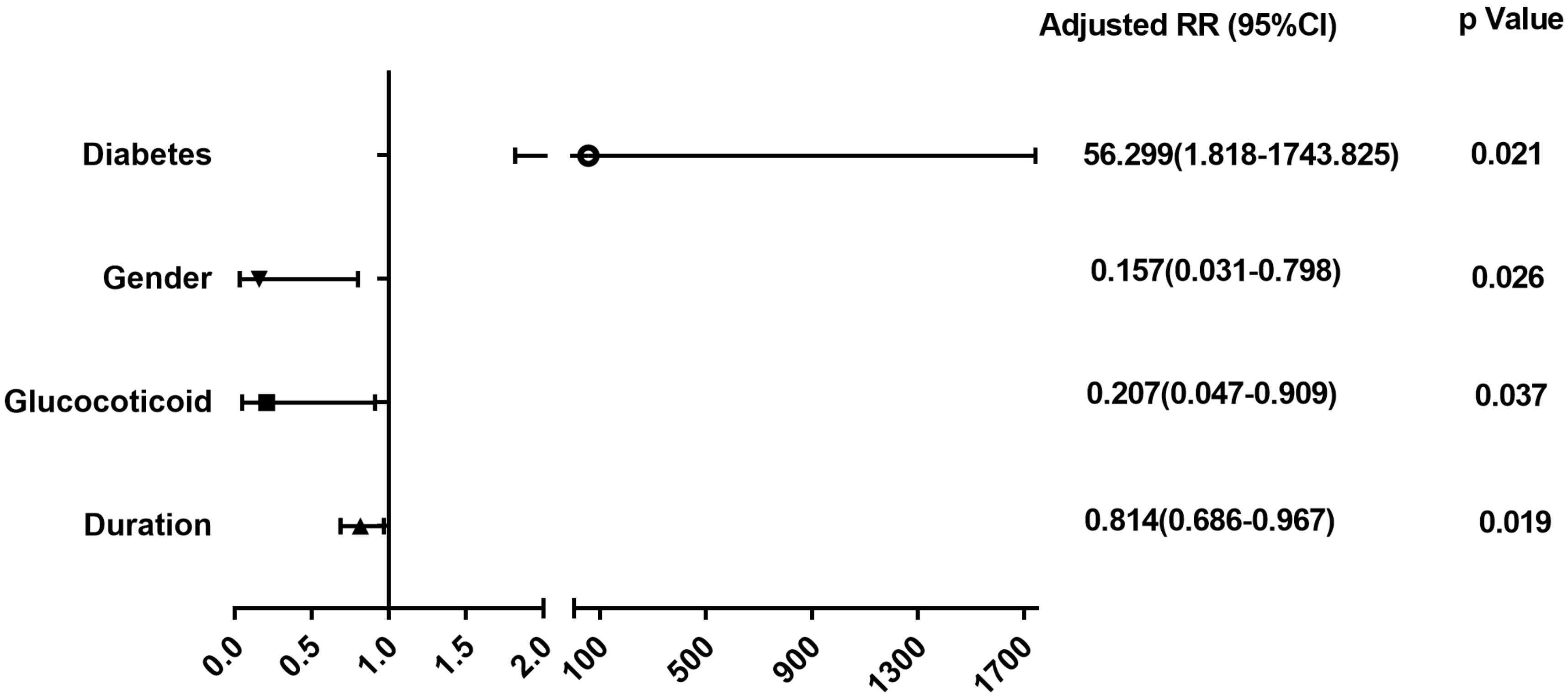

Supplement: Supplementary file 1 [file Image_1.pdf]
