# Supplementary material for: The Incidence Rate and Risk Factors of Malignancy in Elderly-Onset Inflammatory Bowel Disease: A Chinese Cohort Study From 1998 to 2020
Source: Front Oncol. 2021 Dec 9;11:788980. doi: 10.3389/fonc.2021.788980 (PMC8695610; doi:10.3389/fonc.2021.788980)
Supplement: Supplementary file 2 [file Table_1.docx]

Supplementary Material

Supplementary Table1. Incidence cases of malignancy among adult-onset and elderly-onset groups from 1998 to 2008

|  | Adult-onset | | Elderly-onset | |
| --- | --- | --- | --- | --- |
| Year | Cancer cases(N) | Total Patient-years | Cancer cases(N) | Total Patient-years |
| 1998 | 0 | 9.67 | 0 | 0 |
| 1999 | 0 | 12.0 | 0 | 0 |
| 2000 | 0 | 14.5 | 0 | 0.10 |
| 2001 | 0 | 18.0 | 00 | 1.00 |
| 2002 | 1 | 24.2 | 0 | 1.70 |
| 2003 | 0 | 33.9 | 0 | 2.00 |
| 2004 | 0 | 43.0 | 0 | 3.20 |
| 2005 | 1 | 50.6 | 0 | 4.38 |
| 2006 | 1 | 65.5 | 0 | 5.19 |
| 2007 | 1 | 86.6 | 0 | 6.65 |
| 2008 | 2 | 110.8 | 0 | 8.47 |
