# Supplementary material for: The Incidence Rate and Risk Factors of Malignancy in Elderly-Onset Inflammatory Bowel Disease: A Chinese Cohort Study From 1998 to 2020
Source: Front Oncol. 2021 Dec 9;11:788980. doi: 10.3389/fonc.2021.788980 (PMC8695610; doi:10.3389/fonc.2021.788980)
Supplement: Supplementary file 3 [file Table_2.docx]

Supplementary Material

Supplementary Table2. Distribution and incidence rates of malignancy among adult-onset IBD groups

|  | Young-aged onset IBD  (Age 18-40) | | |  | Middle-aged onset IBD  （Age 40-59） | | |  |
| --- | --- | --- | --- | --- | --- | --- | --- | --- |
|  | N(%) Per 1000 PYs[95%CI] | | |  | N(%) | | Per 1000 PYs [95% CI] |  |
| GI malignancies  Colorectal cancer  SBA  Appendiceal mucinous neoplasms  Hepatobiliary malignancy | | 23(50.0)  21(45.7)  0  1(2.17)  1(2.17) | 4.2[2.8-6.3]  3.8[2.5-5.8]  0  0.2[0-1.1]  0.2[0-1.1] | |  | 9(29.0)  6(19.4)  1(3.23)  1(3.23)  1(3.23) | 3.4[1.8-6.5]  2.3[1.1-5.0]  0.4[0.1-2.2]  0.4[0.1-2.2]  0.4[0.1-2.2] | |
| Lung cancer | | 1(2.17) | 0.2[0-1.1] | |  | 4(12.9) | 1.5[0.6-3.9] | |
| Urinary tract malignancy  Hematological malignancy | | 5(10.9)  5(10.9) | 0.9[0.4-2.1]  0.9[0.4-2.1] | |  | 0  2(6.45) | 0  0.8[0.2-2.8] | |
| Thyroid cancer | | 6(13.0) | 1.1[0.5-2.4] | |  | 5(16.1) | 1,9[0.8-4.4] | |
| Genital malignancy | |  |  | |  |  |  | |
| Female breast cancer | | 3(6.52) | 0.5[0.2-1.5] | |  | 2(6.45) | 0.8[0.2-2.8] | |
| Prostate cancer  Uterus malignancy | | 1(2.17)  1(2.17) | 0.2[0-1.1]  0.2[0-1.1] | |  | 0  6(19.4) | 0  2.3[1.1-5] | |
| ovarian cancer | | 0 | 0 | |  | 2(6.45) | 0.8[0.2-2.8] | |
| Others | | 1(2.17) | 0.2[0-1.1] | |  | 1(3.23) | 0.4[0.1-2.2] | |
| Total | | 46(100) | 8.4[6.3-11.2] | |  | 31(100) | 11.8[8.3-16.7] | |
